# Influence of lifestyle factors with the outcome of menstrual disorders among adolescents and young women in West Bengal, India

**DOI:** 10.1038/s41598-023-35858-2

**Published:** 2023-08-01

**Authors:** Shrinjana Dhar, Kousik Kr. Mondal, Pritha Bhattacharjee

**Affiliations:** 1 Environmental Epigenomics Laboratory, Department of Environmental Science, University of Calcutta, 37, Ballygunge Circular Road, Kolkata, West Bengal 700019 India; 2 Department of Zoology, Mugberia Gangadhar Mahavidyalaya, Vidyasagar University, Bhupati Nagar, Purba Medinipur, West Bengal 721425 India

**Keywords:** Molecular biology, Environmental sciences, Diseases, Endocrinology, Pathogenesis, Risk factors

## Abstract

Menstruation is a natural phenomenon for every female, starting from adolescents to menopausal age. Any disturbances in menstrual patterns can eventually affect one’s physical as well as psychological health which in turn hamper the quality of life of women. Several factors including genetic predisposition as well as lifestyle modifications adversely affect normal menstrual patterns. Hence, this study aims to evaluate the prevalence of menstrual disorders among adolescents and young women as well as the associated risk factors. A cross-sectional random survey was conducted from January 2020 to January 2022 in various schools and colleges. A structured questionnaire was prepared which include anthropometric details, demographic information, and lifestyle patterns. The data were extracted for further statistical analysis. In the overall study population, the prevalence of PCOS, Dysmenorrhea, Menorrhagia, Polymenorrhea, Hypomenorrhea and the irregular menstrual cycle was found at 14.14%, 15.14%, 6.29%, 3.70%, 5.16% and 44.83% respectively. The mean BMI of the study population was 19.949 ± 4.801 kg/m^2^ and the mean WHr was 0.872 ± 0.101, indicating a moderate to high risk of metabolic disorder among the study population. Increased BMI, short sleep, and sedentary and vigorous physical activity can contribute to the risk of developing menstrual disorders. Unhealthy food habits are a major risk factor for menstrual disorders. Lifestyle modifications like healthy food habits, sleeping patterns, physical activity, etc. can effectively reduce the risk of menstrual disorders and also cut down the severity of more complex health problems. In-depth biochemical and molecular analysis is required to identify specific biomarkers.

## Introduction

Menstruation is a universal biological phenomenon for any female through which a woman spends 1/5th portion of her reproductive life. While some women go through their monthly periods without fears or minor discomfort, others experience huge physical and emotional symptoms, before and during menstruation, and the Fédération Internationale de Gynécologie et d'Obstétrique (FIGO) termed this disturbance as the Menstrual Disorder^1^. Generally, menstrual disorder includes amenorrhea, abnormal uterine bleeding (menorrhagia, oligomenorrhea, polymenorrhea, hypomenorrhea), dysmenorrhea and premenstrual syndrome, etc. Studies showed that nowadays menstrual disorders are common mostly among adolescents and become less frequent after 3–5 years of menarche^2–4^. With menstruation, lots of myths and misconceptions are still associated to such an extent that it even affects the quality of life, along with other dimensions of life such as education, religious beliefs and health issues^5,6^.

Hitherto there is plenty of research conducted all over the world to explore the prevalence of menstrual disorders and the menstrual pattern across diverse ethnicities. It has been found that menstrual abnormalities were highly influenced by genetic predisposition^7^, lifestyle patterns like dietary habits^8–10^, physical activity^11–13^, sleeping habits^14,15^, environmental exposures^16,17^, etc. However, in developing countries like India, female reproductive health is a big challenging area of research because of lack of awareness, social stigma, etc. Though some significant studies have been done in several parts of India, most scientists have looked into Polycystic ovarian syndrome (PCOS) while other menstrual abnormalities were often unnoticed and even no data have been still accessed in a few parts of the country.

Keeping this background in mind, this study endeavoured to uncover the prevalence of menstrual disorders among adolescents to young adult women in West Bengal, India. Not only this, the collective impacts of multiple risk factors on menstrual patterns have been tried to explain through this research work.

## Result

**Figure 1:**
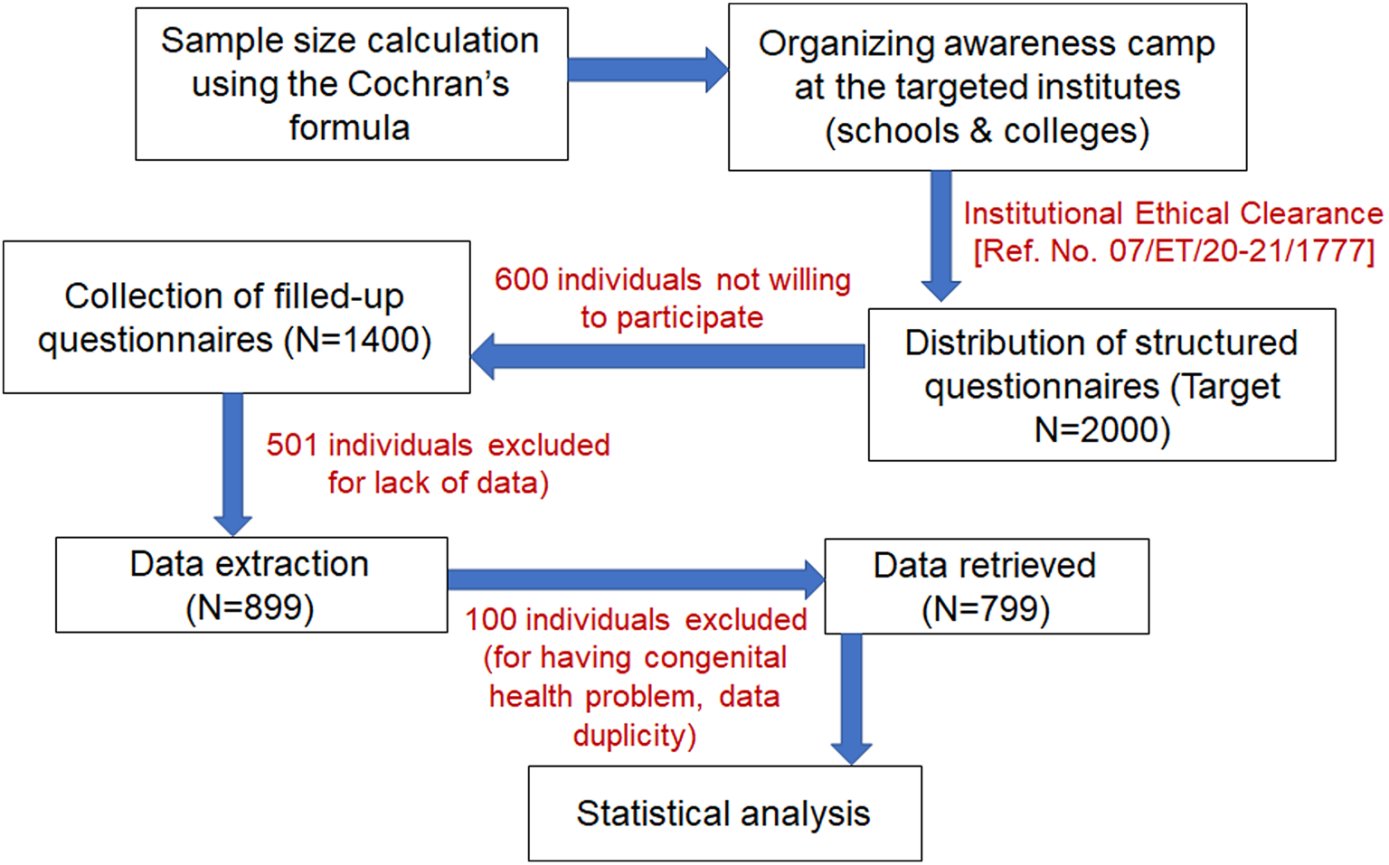
Schematic representation of the study design.

For this epidemiological survey, 7 educational institutes (including schools, colleges, and universities) were approached out of which 4 institutes permitted. A total of 2000 structured questionnaires were administered, 600 individuals declined to participate and 1400 (70%) individuals showed interest to be part of this survey. Of those, 501 questionnaires were rejected due to lack of information and data was extracted from 899 (44.95%) questionnaires. However, 100 data were again excluded due to data duplicity and other congenital health problem, so finally, 799 (39.95%) data were taken into consideration for further statistical analysis (Fig. 1).

**Figure 2:**
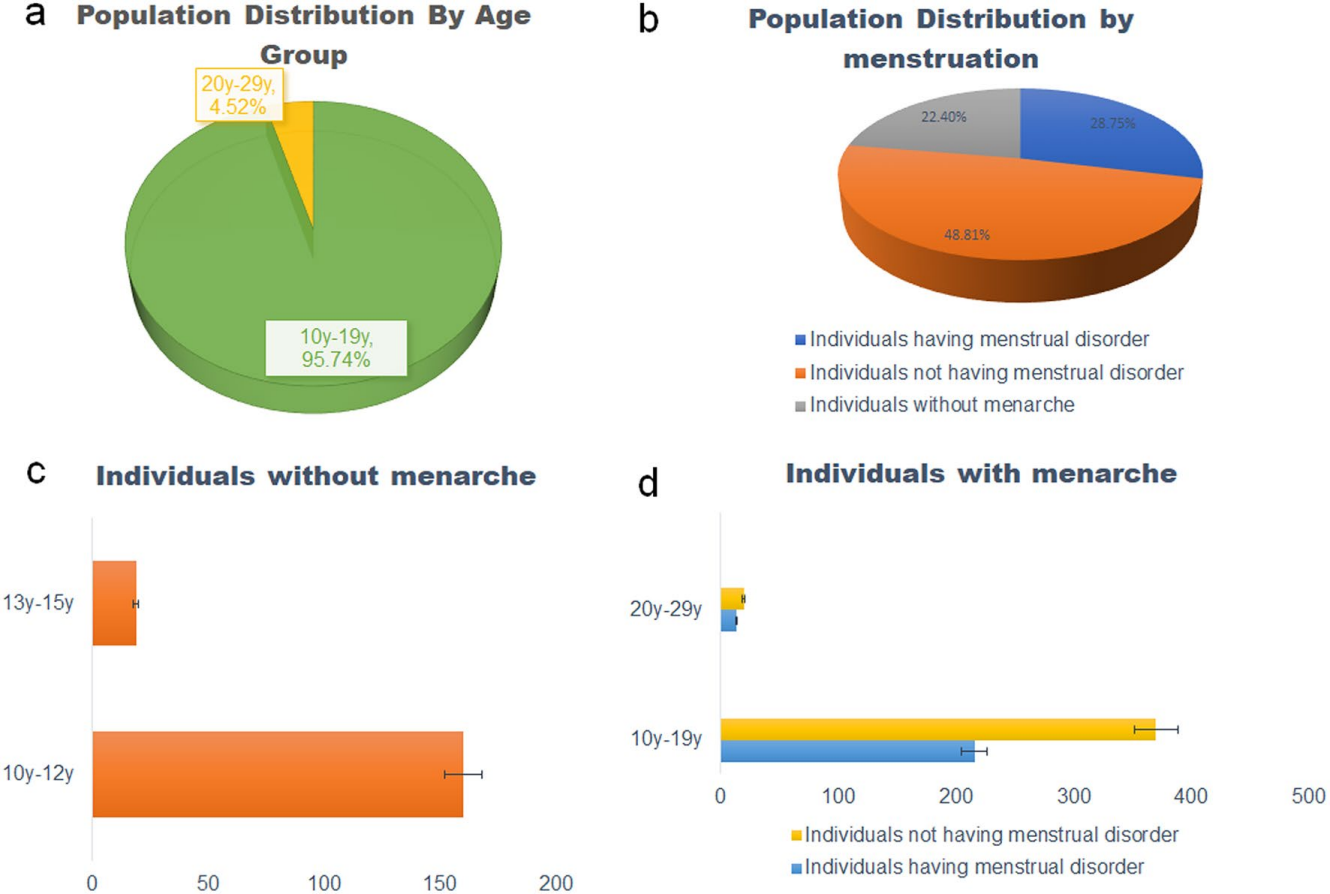
(**a**) Population distribution stratified by age groups; (**b**) Population distribution stratified by menstruation pattern; (**c**) Proportion of individuals without menarche stratified by age groups; (**d**) Proportion of individuals without menarche stratified by age groups.

Table 1 demonstrated the general demographic information of the study population. The median age was 13 years ranging from 10 to 29 years. Overall, 97.37% were Hindu and Muslims accounted for 2.62%; the socio-economic status of the population showed that 46.44%, 34.54%, 11.89% and 6.88% belonged to the lower economic class, lower-middle economy class, upper-middle economy class and upper economy class respectively. The mean BMI of the study population was 19.949 ± 4.801 kg/m^2^ and the mean WHr was 0.872 ± 0.101, indicating a moderate to high risk of metabolic disorder among the study population. The population distribution by age groups was depicted in Fig. 2a and the population distribution by menstruation pattern was shown in Fig. 2b. Among 799, 179 (22.40%) individuals did not achieve menarche at the point of the study and the mean age of that group was 11 ± 1.03 years. Among them, the age of 89.38% of individuals was between 10 and 12 years and the rest are between 13 and 15 years (Fig. 2c). However, individuals who have reached their menarche were categorized into two groups based on their self-reported PCOS and Dysmenorrhea/Endometriosis, i.e., individuals with a menstrual disorder and individuals without a menstrual disorder. The age-wise pattern of menstruation was shown in Fig. 2d.

**Table 1:** Demographic details of the studied population from West Bengal, India (N = 799).

Characteristics	Individuals without menarche (N = 179)		Individuals with menstrual disorder (N = 230)		Individuals without menstrual disorder (N = 390)		*p* value
	Mean (range)	SD	Mean (range)	SD	Mean (range)	SD	
Height (cm)	141.3 (100.5–162)	10.023	152.654 (108–172.72)	9.763	152.993 (100.5–195)	11.109	0.7014
Weight (kg)	36.412 (20–85)	9.311	48.647 (25–89.9)	12.824	46.730 (20–89.6)	10.703	0.0460
BMI (kg/m^2^)	18.492 (8.656–39.850)	4.976	20.908 (10.817–34.723)	5.097	20.051 (7.890–38.642)	4.374	0.0272
Waist (cm)	73.746 (30–165.1)	14.615	78.357 (43–104)	11.072	76.624 (50.8–127)	11.349	0.0643
Hip (cm)	82.872 (35–177.8)	18.267	91.704 (55.88–121.92)	12.036	88.799 (50–130)	12.365	0.0045
Waist–hip ratio (WHr)	0.905 (0.612–1.7)	0.15	0.856 (0.667–1.454)	0.074	0.866 (0.623–1.225)	0.082	0.1290
Age of menarche (years)	N.A	N.A	11.360 (8–15)	1.845	11.553 (9–15)	1.737	0.1921

Characteristics	Individuals without menarche (N = 179)		Individuals with menstrual disorder (N = 230)		Individuals without menstrual disorder (N = 390)		*p* value
	N	(%)	N	(%)	N	(%)	
Religion							
Hindu	170	94.97	223	96.95	385	98.71	0.1254
Muslim	9	5.02	7	3.04	5	1.28	0.1244
Others	0	0	0	0	0	0	0
Socio-economic status							
Lower economy	79	44.13	106	46.08	186	47.69	0.6983
Lower middle class	76	42.45	74	32.17	126	32.30	0.9733
Upper middle class	20	11.17	31	13.47	46	11.79	0.5403
Upper economy	4	2.23	19	8.26	32	8.20	0.9791

**Figure 3:**
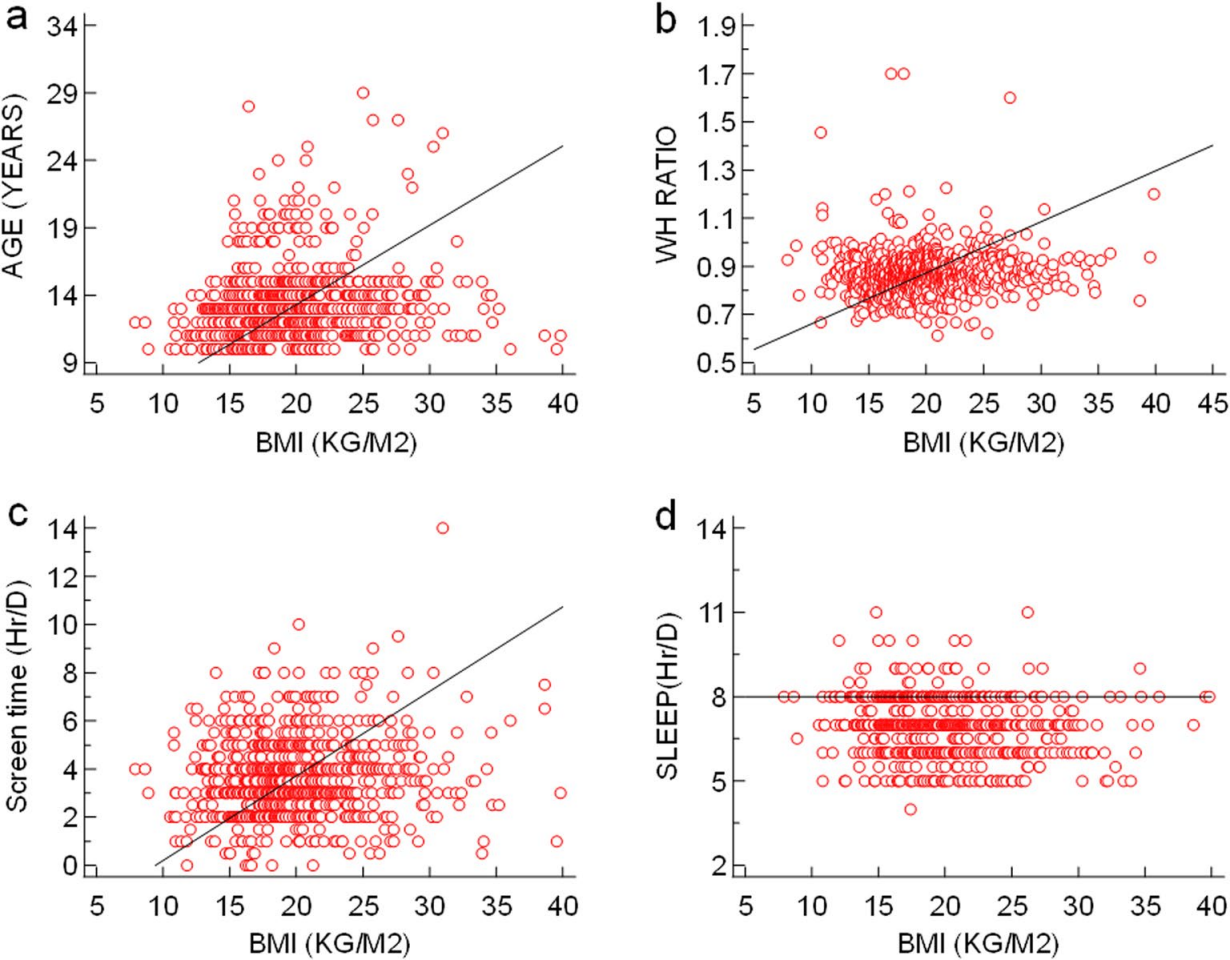
(**a**) Association between age and BMI (r = 0.1427, *p* = 0.0001, 95% CI for r = 0.07412 to 0. 2100); (**b**) Association between WH ratio and BMI (r = 0.06785, *p* = 0.05, 95% CI for r =  − 0.0015 to 0.1366); (**c**) Association between screen time and BMI (r = 0.1166, *p* = 0.001, 95% CI for r = 0.04767 to 0.1845); (**d**) Association between sleep duration and BMI (r =  − 0.1319, *p* < 0.001, 95% CI for r =  − 0.1995 to 0.06300).

**Figure 4:**
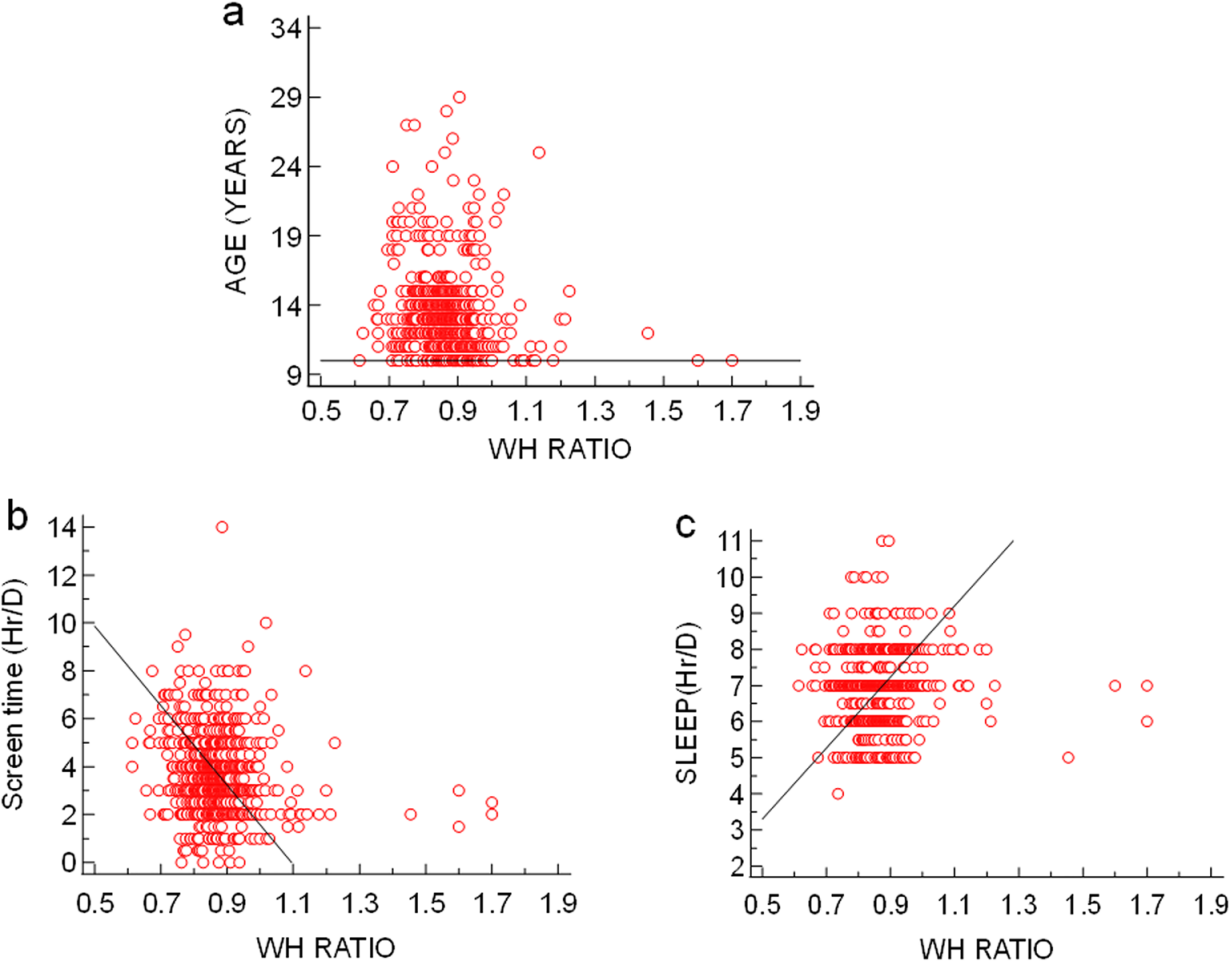
(**a**) Association between age and WH ratio (r =  − 0.1693, *p* < 0.0001, 95% CI for r =  − 0.2359 to − 0.1011); (**b**) Association between screen time and WH ratio (r =  − 0.1210, *p* < 0.001, 95% CI for r =  − 0.1888 to 0.05199); (**c**) Association between sleep duration and WH ratio (r = 0.03506, *p* = 0.3232, 95% CI for r =  − 0.03451 to 0.1043).

**Figure 5:**
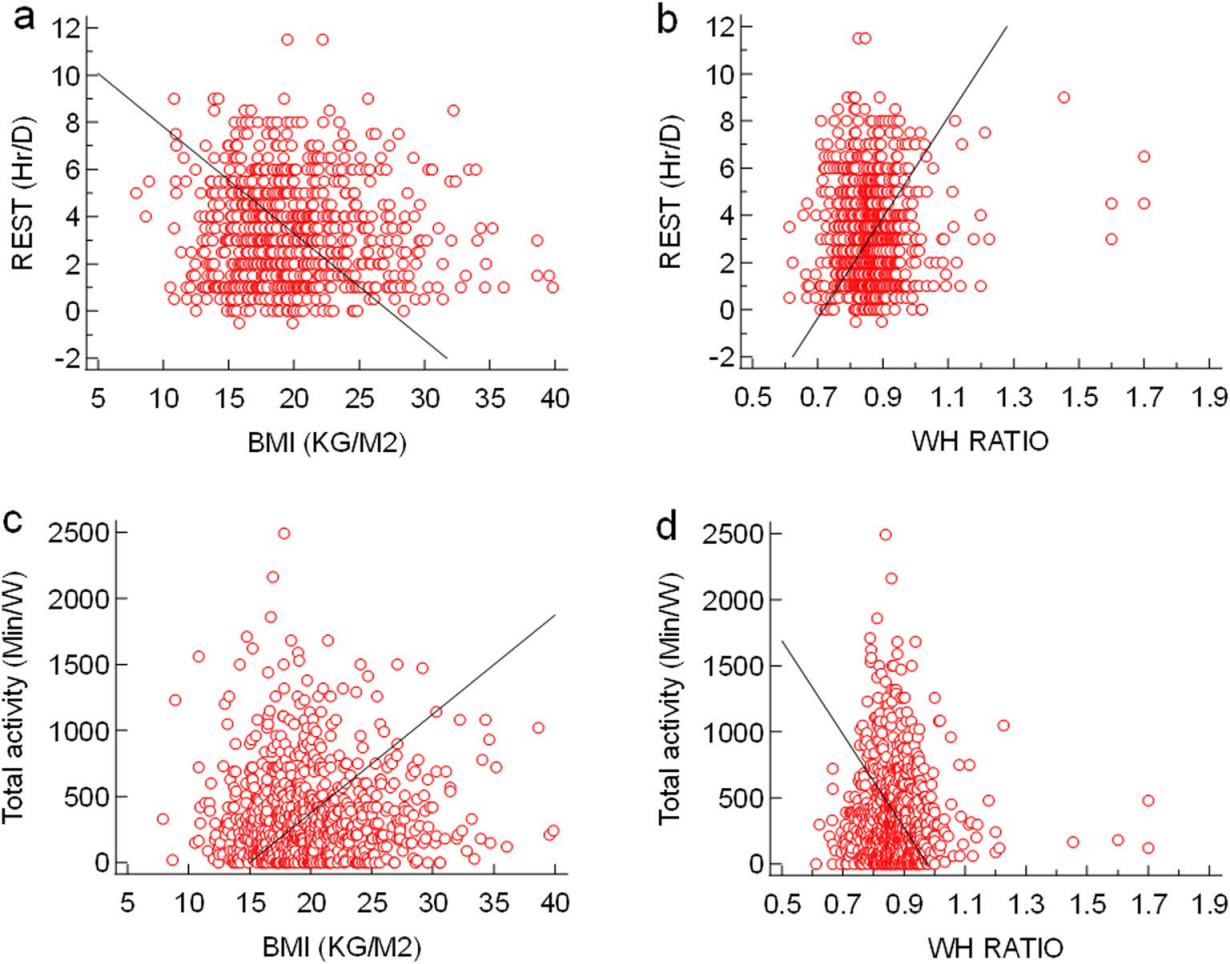
(**a**) Association between rest time and BMI (r =  − 0.05815, *p* = 0.1005, 95% CI for r =  − 0.1270 to 0.01125); (**b**) Association between rest time and WH ratio (r = 0.06918, *p* = 0.0508, 95% CI for r =  − 0.0002261 to 0.1379); (**c**) Association between total physical activity and BMI (r = 0.02536, *p* = 0.4740, 95% CI for r =  − 0.0440 to 0.0945); (**d**) Association between total physical activity and WH ratio (r =  − 0.0217, *p* = 0.5397, 95% CI for r =  − 0.0910 to 0.0477).

**Figure 6:**
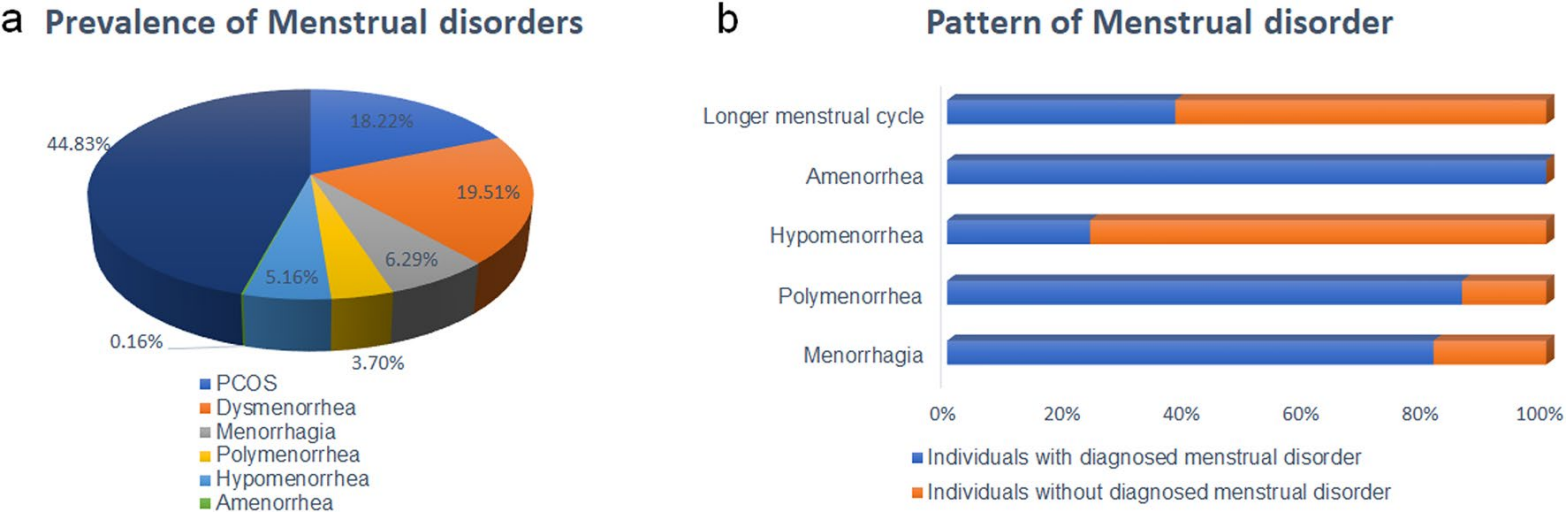
(**a**) Pie diagram depicting the prevalence of menstrual disorders; (**b**) Patterns of menstrual disorders in the study population.

The correlation coefficient study showed that a highly significant positive association was observed between BMI and age, screen time, and WHr while a negative association was observed between BMI and sleep duration (Fig. 3). WHr was significantly inversely associated with age and screen time (Fig. 4). The study was conducted during the COVID-19 pandemic while the overall population was following sedentary lifestyle and screen time was usually higher because they were dependent upon mobile/laptop/computer for their study/work. Therefore, leisure time became shortened. On the other hand, total physical activity of the study population was majorly dependent upon walking and this (intensity of physical activity) was not enough sufficient to reduce BMI. Henceforth, avery weak positive association was observed between BMI and total physical activity as well as WHr and leisure time, at the same time, weak inverse association was found between BMI and leisure time as well as WHr and total physical activity (Fig. 5). The prevalence of menstrual disorders (PCOS and/or Dysmenorrhea/Endometriosis) in the study population was found to be 28.78%. The demographic pattern showed that the mean body weight (kg), BMI (kg/m^2^) and hip circumference (cm) (48.647 ± 12.824 *vs.* 46.730 ± 10.703, 20.908 ± 5.097 *vs.* 20.051 ± 4.374 and 91.704 + 12.036 *vs.* 88.799 ± 12.365 respectively) in individuals with a menstrual disorder were significantly higher (*p* < 0.05) compared to those without a menstrual disorder. The average menstrual interval among individuals with a menstrual disorder was 35.417 ± 10.404 days while that of 29.369 ± 4.261 days among individuals without a menstrual disorder. The mean duration of a period was 5.986 ± 2.118 days in individuals with a menstrual disorder and 5.3 ± 1.486 days in individuals without a menstrual disorder. However, the prevalence of all menstrual-related issues in the study population has been represented in Fig. 6a. The most interesting fact has been found that individuals who are not diagnosed with either PCOS or Dysmenorrhea/Endometriosis, also showed similar symptoms (like Menorrhagia, Polymenorrhea, Hypomenorrhea, and Longer menstrual cycle) as shown by individuals diagnosed with PCOS and/or Dysmenorrhea/Endometriosis (Fig. 6b). The prevalence of PCOS, Dysmenorrhea, Menorrhagia, Amenorrhea, Polymenorrhea, Hypomenorrhea and the irregular menstrual cycle was found to be 49.13%, 52.60%, 12.17%, 0.43%, 7.82%, 2.17% and 32.17% respectively among those who are diagnosed already. However, for those who are not diagnosed or fall into the control category, among them, the prevalence of Menorrhagia, Polymenorrhea, Hypomenorrhea and the irregular menstrual cycle was 2.82%, 1.28%, 6.92% and 52.30% respectively. It was estimated that ~ 67% (66.92%) population is still not under any diagnosis or is unaware of the problem. This is also reflected in the use of sanitary napkins, ~ 37% (37.58%) of the population are not using sanitary napkins.

Table 2 described the association of multiple risk factors with the incidence of menstrual disorders among adolescents and young women. It has been found that individuals of age between 20 and 29 years were almost 2 times (OR = 1.199, *p* value = 0.6129, 95% CI 0.5935 to 2.4226) more prone to have a menstrual disorder. On the other hand, overweight individuals were at ~ 2 times more risk (OR = 1.943, *p* < 0.05, 95% CI 1.1781–3.2044) to get menstruation-related disorders than obese (OR = 1.737,* p* value = 0.1688, 95% CI 0.7911–3.8154). Short sleep can increase > 1.5 times more risk to get a menstrual problem (OR = 1.623, *p* < 0.05, 95% CI 1.0015–2.6326). It has been found that sedentary as well as moderate and vigorous physical activity may increase the risk of menstrual disorders whereas low physical activity can be a protective factor (OR = 0.662, *p* < 0.05, 95% CI 0.4728–0.9271) against the risk of a menstrual problem. Data suggested that gastro-intestinal issues (OR = 3.806, *p* < 0.0001, 95% CI 2.0736–6.9863), tiredness/dizziness (OR = 1.548, *p* < 0.05, 95% CI 1.1077–2.1631), frequent headache (OR = 1.682, *p* < 0.005, 95% CI 1.2069–2.3454) and anaemia (OR = 3.226, *p* < 0.005, 95% CI 1.4627–7.1168) are significantly associated with the outcome of menstrual disorders.

**Table 2:** Risk factors for menstrual disorders among the study population from West Bengal, India.

Variables	Total population (N = 799)		Individuals with diagnosed menstrual disorder (N = 230)		Individuals without diagnosed menstrual abnormality (N = 390)		Odds ratio (OR)	*p* value [95% confidence interval]
	N	%	N	%	N	%		
Age (years)								
Group-A (10–19)	765	95.74	216	93.91	370	94.87	0.834	0.6129 [0.4128–1.6850]
Group-B (20–19)	34	4.25	14	6.08	20	5.12	1.199	0.6129 [0.5935–2.4226]
BMI (kg/m^2^)								
Underweight (< 18.5)	340	42.55	81	35.21	151	38.71	0.860	0.3844 [0.6132–1.2073]
Normal (18.5–25)	346	43.30	101	43.91	192	49.23	0.807	0.2004 [0.5819–1.1202]
Overweight (25–29.9)	85	10.63	36	15.65	34	8.71	1.943	0.0093 [1.1781–3.2044]
Obese (> 29.9)	30	3.75	13	5.65	13	3.34	1.737	0.1688 [0.7911–3.8154]
WH ratio								
Normal (< 0.85)	311	38.92	96	41.73	160	41.02	1.029	0.8616 [0.7398–1.4336]
Moderate risk (0.85–0.90)	247	30.91	91	39.56	115	29.48	1.565	0.0103 [1.1116–2.2047]
High risk (> 0.90)	240	30.03	43	1.30	115	29.48	0.549	0.0031 [0.3699–0.8175]
Consumption of beverage								
Only tea	210	26.28	61	26.52	105	26.92	0.979	0.9132 [0.6778–1.4162]
Only coffee	47	5.88	18	7.82	21	5.38	1.491	0.2290 [0.7774–2.8631]
Tea + coffee	301	37.67	88	38.26	141	36.15	1.094	0.5995 [0.7815–1.5325]
Sleep duration (hours/day)								
Short sleep (< 6)	79	9.88	36	15.65	40	10.25	1.623	0.0493 [1.0015–2.6326]
Normal sleep (6–8)	685	85.73	190	82.60	337	86.41	0.747	0.2013 [0.4776–1.1685]
Excessive sleep (> 8)	34	4.25	4	1.73	13	3.34	0.513	0.2485 [0.1654–1.5933]
Physical activity (min/week)								
Sedentary (< 40)	121	15.14	37	16.08	56	14.35	1.143	0.5607 [0.7280–1.7959]
Low activity (40–600)	526	65.83	133	57.82	263	67.43	0.662	0.0164 [0.4728–0.9271]
Moderate (600–1200)	137	17.14	54	23.47	61	15.64	1.654	0.0159 [1.0989–2.4920]
Vigorous (> 1200)	31	3.88	10	4.34	16	4.10	1.062	0.8830 [0.4739–2.3823]
Presence of health problems								
Gastro-intestinal issue	58	7.25	34	14.78	17	4.35	3.806	< 0.0001 [2.0736–6.9863]
Tiredness/dizziness	266	33.29	101	43.91	131	33.59	1.548	0.0105 [1.1077–2.1631]
Frequent headache	283	35.41	109	13.64	136	34.87	1.682	0.0021 [1.2069–2.3454]
Anaemia	29	3.63	18	7.82	10	2.56	3.226	0.0037 [1.4627–7.1168]
Arthritis	2	0.25	0	0	1	0.25	0.563	0.7256 [0.0228–13.8850]
Diabetes	0	0	0	0	0	0	0	0
Menstrual health								
Only PCOS	59	7.38	59	25.65	0	0	270.159	0.0001 [16.6549–4408.2530]
Only dysmenorrhea/endometriosis	68	8.51	67	29.13	0	0	322.431	< 0.0001 [19.8420–5239.4920]
PCOS + dysmenorrhea/endometriosis	55	6.88	55	23.91	0	0	246.982	0.0001 [15.1699–4021.1661]
Menorrhagia	39	4.88	28	12.17	11	2.82	4.775	< 0.0001 [2.3292–9.7926]
Amenorrhea	1	0.12	1	0.43	0	0	5.104	0.3188 [0.2071–125.8327]
Polymenorrhagia	23	2.87	18	7.82	5	1.28	6.537	0.0003 [2.3933–17.8589]
Hypomenorrhea	32	4	5	2.17	27	6.92	0.298	0.0145 [0.1134–0.787]
Menstrual cycle (day)								
Normal (25–30)	353	44.18	77	33.47	276	70.76	0.207	< 0.0001 [0.1465–0.2950]
Probable risk (31–35)	278	34.79	74	32.17	204	52.30	0.432	< 0.0001 [0.3077–0.6080]
Oligomenorrhea (> 35)	90	11.26	90	39.13	0	0	503.064	< 0.0001 [31.0169–8159.2116]

The general food habits of the study population and their influence on the menstrual disorder can be estimated and depicted in Supplementary Table S1 online. Results showed that more than 5 cups per day of cooked rice intake can increase ~ 7 times risk (OR = 6.991, *p* < 0.05, 95% CI 1.4716–33.2114), frequent soy product intake can increase approximately 2 times more risk (OR = 1.856, *p* < 0.0005, 95% CI 1.3348–2.5812), no egg intake (OR = 1.82, *p* < 0.05, 95% CI 1.1042–3.0016) or no sugar intake (OR = 1.435, *p* = 0.05, 95% CI 0.9862–2.0881) can also increase risk of menstrual abnormalities. Also, it has been found that intake of fried food items, processed foods and packaged fruit juice may be associated with the risk of several menstrual discomforts. In contrast to this, the risk of the outcomes can be significantly drop down by stopping soy product intake (OR = 0.436, *p* < 0.0001, 95% CI 0.3085–0.6187) and daily egg intake (OR = 0.596, *p* < 0.05, 95% CI 0.3687–0.9638) as well as fish/chicken (OR = 0.629, *p* < 0.05, 95% CI 0.4076–0.9708) consumption. In addition, menstrual abnormalities can be greatly influenced by the usage of multiple synthetic cosmetic materials as described in Supplementary Table S2 online.

## Discussion

Menstrual disorders are referred to as a combination of one or more deleterious symptoms of menstrual patterns that can have a greater impact on the daily living of women and contribute to several other morbid conditions like infertility. Therefore, this present study attempted to assess the prevalence of menstrual abnormalities among adolescents to young adult women and to understand the cumulative role of multiple risk factors (like socio-demographic profile, dietary habits, etc.).

An earlier study reported that the mean age of menarche in Central Indian girls was 13.5 years^11^, whereas the mean age of menarche in the study population was 11.4 years which has been earlier found by Żegleń et al.^19^. Meanwhile, 10.61% of girls have delayed menarche in the study population. In the middle-east population, the prevalence of menstrual disorder was found to be more than 50%^10,20^ while it was about 22.7% in the Southeast Asian population^2^. In an Italian adolescent population, the prevalence of Dysmenorrhea, Polymenorrhea, Oligomenorrhea, Hypomenorrhea, Menorrhagia and the irregular menstrual cycle was 6.2%, 3%, 3.4%, 3.2%, 19% and 9% respectively^3^. In the Iranian adult population, 41% of respondents suffered from Dysmenorrhea and 22.1% suffered from irregular menstrual cycles^10^. In another study, incidences of Irregular Menstruation, Abnormal vaginal bleeding, Amenorrhea, Menorrhagia, Dysmenorrhea and Premenstrual Symptoms were 27%, 9.3%, 9.2%, 3.4%, 89.7% and 46.7% respectively^20^. In Singaporean teenagers, 23.1% reported having irregular menstrual cycles and Oligomenorrhea, Polymenorrhea and Amenorrhea prevalence were 17.5%, 2% and 1.2% respectively^2^. In India, 30.48% and 56.15% of adolescents were suffering from irregular menstrual cycles and Dysmenorrhea respectively^11^; 61% and 16.4% of adolescents were suffering from Oligomenorrhea and primary Amenorrhea respectively^21^. It was observed that the prevalence of Dysmenorrhea, Premenstrual Symptoms and irregular menstrual cycles were 71.2%, 70.2% and 14.8% respectively among young adult women^22^ whereas that of Dysmenorrhea and Menorrhagia were 45% and 17% respectively among other adult women^23^. In West Bengal, a prospective single-centre study reported the percentage of PCOS patients was 75.47% among the age group of 13 to 45 years^24^ whereas a college-based survey reported that 28% of the respondents were at risk of developing PCOS^18^. In our study, the prevalence of menstrual disorders (PCOS and/or Dysmenorrhea) was about 28.78% among adolescents and young women. In the overall study population, the prevalence of PCOS, Dysmenorrhea, Menorrhagia, Polymenorrhea, Hypomenorrhea and the irregular menstrual cycle was found at 14.14%, 15.14%, 6.29%, 3.70%, 5.16% and 44.83% respectively.

Among the most discussed risk factors for menstrual disorders, BMI and WH-ratio are often the top-ranked. It has been found that women with PCOS had a higher BMI than control women^13,18^. Other findings revealed that a proportion of women with menstrual disorders have significantly higher BMI (overweight and obese) than normal women^10^ and the increase in BMI is associated with abnormality in menstrual cycle length, period duration, and blood loss^25^. Contrasting to this, malnutrition due to the eating disorder may often be associated with Oligo/Amenorrhea among adolescents and young women^26^. Adolescents having BMI < 18.5 showed more symptoms of menstrual disorders^27–29^. However, Bhattacharya et al.^30^ suggested that WH-ratio can be a better index for PCOS than BMI. A higher waist and hip circumference were observed among the PCOS females than their non-PCOS females, WHr appeared to be significantly higher among the PCOS group^18^. Increased WHr was associated with periodic blood loss only whereas increased Waist-to-height ratio was associated with abnormal cycle length and period duration, but not menstrual blood loss^25^. In our study, both BMI and WHr were significantly high among individuals with the menstrual disorder than those without the disorder. Though there is no significant correlation exists between BMI and WHr in the present sample, indicating the occurrence of metabolic disturbances is independent of body weight.

No such reports have been available on the association between physical activity and menstrual disorders. It was found that girls involved in vigorous sporting activity have a significantly higher age of menarche^11^. Little or no physical activity may be a risk factor for developing PCOS^9,31^. Along with the frequency of physical activity, the risk of PCOS also depends upon the intensity of exercise^12^. Strong physical activity and eating disorders often lead to oligomenorrhea in adolescents, though physical activity decreases with an increase in age^3^. Similar findings, i.e., sedentary as well as vigorous physical activity can be a risk factor for the rising menstrual disorder. Not only physical activity, but the sleeping pattern also plays an important role in proper metabolism and menstrual activity. A cross-sectional study of the Australian population reported that adverse sleep symptoms like severe tiredness, difficulty sleeping and restless sleep were significantly associated with PCOS^14^. Other studies showed an association between short sleep duration, poor sleep quality, fatigue, stress and depression with heavier bleeding and menstrual cycle irregularity^29,32^. Herein our study also found an association between short sleep and the risk of developing menstrual disorders.

Dietary habits are one of the most important lifestyle parameters associated with almost every disease. In a study of Iranian adolescent girls, it has been observed that skipping meals (especially breakfast) and consuming unhealthy foods such as fast food, soft drinks, sweets and junk food may contribute to the development of PCOS^9,31^. Specifically, high loadings of carbohydrates, animal protein, fat, cholesterol, saturated fatty acid, sodium, biotin, copper, iron, fluoride, zinc, and calcium can significantly increase the risk of PCOD^33^. On the other hand, menstrual disorders like irregular menstruation, painful menstruation, and Premenstrual syndrome (PMS) were found to be significantly associated with a high intake of calories, proteins, carbohydrates, and total fat^10^. Skipping breakfast and frequent junk food intake were associated with Dysmenorrhea among adolescents^28^. In our study,consumption of excess cooked white rice, soy foods and unhealthy dietary intakes like fried food items, processed foods and packaged fruit juice could be an important contributing factor to increased risk of menstrual disorders. If a healthy dietary pattern would be made (keeping ‘Indian scenario’ in mind) based on Supplementary Table S1 (example, sufficient water intake + daily 1–2 cups of rice + 1–2 pcs. Roti + daily 1pc. egg + daily 1 pc. Fish/chicken + no fried foods + no soy products + no processed foods + no packaged drink), the risk of menstrual disorder can be reduced. In support to this statement, our data also showed that individuals having no menstrual disorders (4.87%) consume this healthy food choice compared to individuals having menstrual disorders (1.73%). This may indicate the importance of food selection in the manifestation of menstruation-related disorders.

Menstrual disorders not only impact female reproduction or infertility but it is also associated with other health factors. Zeru et al.^29^ reported that anaemia is one of the associated factors with menstrual disorders. Thyroid dysfunction was often found to be associated with menstrual disorders^21,24^. About 24% of teenagers were reporting school absenteeism owing to menstrual disorder^2^ and a good proportion of adults also reported absenteeism in their workplaces^22,23^. Our study revealed that gastrointestinal problems, dizziness/tiredness, frequent headaches and anaemia were significantly associated with menstrual disorders. Hence, the overall study was reflecting the hindrance of quality life of adolescents and young women. However, during the survey, few individuals confronted that they have a parental history of hypertension, cardiovascular disease, anaemia, thyroid dysfunction, type 2 diabetes mellitus, gynaecological issues, etc., therefore it is indeed important to analyze the disease outcomes correlating with family history.

The strength of the present study is a good sample size. It is way too interesting to work on the human population by being aware of them and convince to participate in the survey process. Though it is the twenty-first century, in developing countries like India where female health issues are often neglected or criticized, it was more difficult to complete the survey because people are usually not willing to discuss menstruation-related information with a non-clinical person. On the other hand, studies so far predominantly focus on either PCOS, endometriosis or any gynaecological cancers, but limited studies have been done on menstrual disorders, so a large group of women are still unaware of the menstrual problems. Early detection of the symptoms of menstrual disorders can prevent women from getting complex health issues like gynaecological cancers (ovarian cancer, breast cancer, etc.) or infertility. As mentioned earlier, menstrual disturbances affect the overall quality of life of a woman (like rapid mood swings, skin issues, painful cramps, etc.). It is often found that students cannot attend schools and colleges during those days, employees take leave from their offices, etc. which ultimately a loss for nations in terms of economy. Henceforth, this study may provide a scenario of menstrual disorders among adolescents and young women so that the Government health sector may take action against early detection, awareness of menstrual hygiene, etc. In this regard, Government may implement some health scheme that can provide a low-cost diagnostic facility for the general population. Also, nation-wide awareness camp needs to be conducted to aware people about their reproductive health and hygiene. Along with this, through National Education Policy (NEP), menstrual health education must be initiated at school before students reach puberty so that girls can aware of their health issues and can overcome the fear or social stigma at a very early age. Schools, colleges, NGOs, private or Government health care sectors may provide sanitary napkins, menstrual cups, tampons, etc. along with training of their proper use. Nonetheless, this study also gives an idea of several risk factors for menstrual disorders and some preventive measures can be obtained by modifying the probable lifestyle factors.

Like every other research work, our study also has limitations. First of all, the total survey was conducted based on self-reporting, so there is a chance of false information (may be due to unwillingness or unawareness) persist. The study was conducted during the pandemic situation, therefore rejection from the institutions was quite obvious (due to fear of spreading infection). Due to the fund crisis, only a questionnaire-based survey was possible to conduct while biochemical analysis of individuals could strengthen the results more.

In conclusion, menstrual disorders refer to a combination of symptoms of uterine bleeding, which can adversely affect the dimension of the life of women. The present study provides the prevalence of menstrual disorders among adolescents and young women as well as highlights the modifiable factors that may contribute to the risk of menstrual disorders. Accumulating the observed results from the study, it can be suggested that detection of any menstrual abnormalities can possible at a very early stage or the risk can be ameliorated by changing the lifestyle patterns. But, in-depth biochemical analysis, as well as genetic-epigenetic studies, are required to identify the biomarkers of each menstrual problem. Government should take initiatives like policy-making in the health and education sectors to aware people and implementation of proper treatment strategies to cut down the economic burden of the countries.

## Methodology

### Selection of study population

The present study was conducted in Kolkata, one of the four metropolitan cities in India, and its peri-urban area. Kolkata has situated on the east bank of the River Ganga in the state of West Bengal. According to the Census 2011, the population of West Bengal was 91,276,115 and the sex ratio and the literacy rate were 950 and 76.26 (the child sex ratio was 956) whereas the population of the Kolkata metropolitan area was 14,035,959 and the sex ratio was 935 (the child sex ratio was 947). It was estimated that the population of children (0–6 years) was 1,158,543 and among them, 563,573 was female population. The Kolkata metropolitan population is of mixed nature with people of different religions, castes, and socioeconomic levels. The reason behind selecting this particular zone was its diversified lifestyle patterns among individuals.

### Sample size and study design

The total sample size was calculated using Cochran’s formula Z^2^ P (1−P)/d^2^, assuming the prevalence of PCOS as 28%^18^ and the attrition of the subjects at any stage of the study due to unavoidable reasons of 5%, a possible error was also taken. As per the calculation, a minimum of 310 sample size should be considered for the study to achieve the power of 80%, while approximately 2000 sample size was initially targeted for the present study. A cross-sectional random survey approved by the Institutional ethical committee (Ref. No. 07/ET/20–21/1777) was conducted from January 2020 to January 2022. Since the target age group was 10 to 30 years (Considering the average age of menarche in Bengali girls is 11.8 years according to^19^), this study was mainly done in schools and colleges. Beside this, participants who are married or unmarried and under any medications were included whereas transgender, pregnant or conceived already and individuals having any congenital health issue were strictly excluded for this study.Before conducting the survey, all individuals were given the personal and societal relevance of the study as well as informed consent was obtained from each participant. In the case of the adolescent group, consent from parents and verification of the self-stated information were also done. The detailed methodology has been schematically represented in Fig. 1.

### Data collection

A structured questionnaire (Supplementary Data S3 online) was prepared which include anthropometric details with an emphasis on demographic information (Religion, Socioeconomic status by monthly salary), physical activity (including walking, jogging, yoga, sports, aerobics/dance), dietary pattern (Sugar intake, Processed food intake, etc.), Screen time (include TV viewing, Mobile and Computer usage timing), Sleep duration, general health issues (include Gastro-intestinal problem, Tiredness, Headache/Bodyache, Anaemia, Arthritis, Diabetes, PCOS if diagnosed by expert clinicians and endometriosis if diagnosed), information regarding menstruation (Age when 1st menstruation started, in general, or how many days ago the last period occurs, how many days the bleeding occurs, how the flow of menses like low or moderate or heavy bleeding, whether they use sanitary napkins or not), and exposure to synthetic cosmetics products (include Talcum powder, Body lotion, Sunscreen lotion, Deodorant, Lipstick, Body soap, Shampoo, Body oil, and Hair oil). Participants were asked to fill up the questionnaire based on the recall method and anthropometric measures were taken by our trained research team The submitted questionnaires were thoroughly scrutinized for analysis based on complete data and those without duplicity. The data were entered into Microsoft Excel 2016 for further analysis.

For demographic details, individuals were grouped into 2 age categories, i.e., Group-A (10–19 years) and Group-B (20–29 years). The socio-economic status was graded as the Lower economic class (income < INR 15,000 per month), Lower middle class (income ≥ INR 15,000 ≤ INR 30,000 per month), Upper middle class (income ≥ INR 30,000 ≤ INR 50,000 per month) and Upper economic class (income > INR 50,000 per month). In anthropometric details, height, weight, waist circumference, and hip circumference were considered. So, the BMI was calculated as weight (in kg) divided by height (in m) squared and was categorized based on WHO recommended standards (Normal weight: 18.5 ≥ BMI ≤ 25 kg/m^2^; Underweight: < 18.5; Pre-obesity/Overweight: 25 kg/m^2^ ≥ BMI ≤ 29.9 kg/m^2^; and obese: BMI ≥ 30 kg/m^2^). The waist-hip ratio was differentiated as Normal (≤ 0.85), Moderate risk (≥ 0.85 ≤ 0.90), and high risk (≥ 0.90) of metabolic complications.

In assessing lifestyle patterns, Water intake has been measured in Litre per day and the use of synthetic chemicals has been recorded as Days per week. Consumption of tea, coffee intake, daily fruit-eating, and presence of any disease has been expressed in binary form, i.e., 0 for NO and 1 for YES. Sleep duration has been assessed as short sleep (≤ 6 h/day), normal sleep (6–8 h/day), and excessive sleep (≥ 8 h/day) as followed by^14,15^. The level of physical activity was stratified as Sedentary (< 40 min/week), Low activity (40–600 min/week), Moderate activity (600–1200 min/week), and Extreme/vigorous activity (> 1200 min/week). Therefore, the leisure time (hours/day) has been calculated by the formula: {24 − (total screen time + working hours + sleep)}.

Categorization of menstrual irregularities was classified as Normal (25–30 days), Possible risk (31–35 days), High risk/Oligomenorrhea (> 35 days), and Amenorrhea (no period > 90 days); however, a menstruation-related disorder like Hypomenorrhea (Low volume bleeding, < 3 days, or both), Dysmenorrhea (painful cramps during menstruation), Polymenorrhea (Occurrence of period < 21 days), Menorrhagia/Endometriosis (Heavy bleeding for > 7 days). The number of days of menstruation as 3–7 days and > 7 days has been considered Normal and Prolonged periods respectively.

### Statistical analysis

Data have been expressed in Mean ± SD as well as percentages (%) values depending on the analysis criteria. Calculations and graphs were produced using MedCalc® Statistical Software v20.115 (MedCalc Software Ltd, Ostend, Belgium) and Microsoft Excel 2016 (Washington, USA). The odds ratio was calculated to assess the influence of environmental determinants on disease outcomes. Two-tailed student t-test was performed to validate the significant difference between the two groups of the population.

### Ethical approval

This study was performed in line with the principles of the Declaration of Helsinki. Approval was granted by the ethical committee of the University of Calcutta (07/ET/20-21/1777).

### Consent to participate

Written informed consent was obtained from all the individual participants and/or their parents included in the study.

## Supplementary Information

Supplementary Information.

## Data Availability

The datasets generated during and/or analysed during the current study are available from the corresponding author on reasonable request.
