# Supplementary material for: Influence of lifestyle factors with the outcome of menstrual disorders among adolescents and young women in West Bengal, India
Source: Sci Rep. 2023 Aug 1;13:12476. doi: 10.1038/s41598-023-35858-2 (PMC10393940; doi:10.1038/s41598-023-35858-2)
Supplement: Supplementary file 1 — Supplementary Information. [file 41598_2023_35858_MOESM1_ESM.docx]

**Supplementary Table S1** Dietary habits of the study population

| **Variables** | **Total population**  **(N = 799)** | | **Individuals with diagnosed menstrual disorder**  **(N = 230)** | | **Individuals without diagnosed menstrual abnormality**  **(N = 390)** | | **Odds ratio (OR)** | ***p-*value [95% Confidence Interval]** |
| --- | --- | --- | --- | --- | --- | --- | --- | --- |
|  | **N** | **%** | **N** | **%** | **N** | **%** |  |  |
| Daily water intake (Litre) | | | | | | | | |
| <2 | 433 | 54.19 | 46 | 20 | 62 | 15.89 | 1.322 | 0.1941 [0.8673 - 2.0169] |
| 2-3 | 534 | 66.84 | 155 | 67.39 | 270 | 69.23 | 0.918 | 0.6338 [0.6475 - 1.3030] |
| >3 | 111 | 13.89 | 29 | 12.60 | 58 | 14.87 | 0.825 | 0.4337 [0.5116 - 1.3333] |
| Rice intake (Cups/Day) | | | | | | | | |
| No | 5 | 0.62 | 2 | 0.86 | 2 | 0.51 | 1.701 | 0.5962 [0.2381 - 12.1639] |
| <2 | 601 | 75.21 | 162 | 70.43 | 301 | 77.17 | 0.704 | 0.0627 [0.4871 - 1.0188] |
| 2-5 | 182 | 22.77 | 58 | 25.21 | 85 | 21.79 | 1.21 | 0.3288 [0.8253 - 1.7739] |
| **>5** | **11** | **1.37** | **8** | **3.47** | **2** | **0.51** | **6.991** | **0.0144 [****1.4716 - 33.2114]** |
| Roti/chapati intake (Pcs./Day) | | | | | | | | |
| No | 114 | 14.26 | 36 | 15.65 | 55 | 14.10 | 1.13 | 0.5985 [0.7165 - 1.7831] |
| <2 | 516 | 64.58 | 147 | 63.91 | 254 | 65.12 | 0.948 | 0.7598 [0.6748 - 1.3327] |
| 2-5 | 161 | 20.15 | 42 | 18.26 | 78 | 20 | 0.893 | 0.5966 [0.5892 - 1.3553] |
| >5 | 8 | 1 | 5 | 2.17 | 3 | 0.76 | 2.866 | 0.1520 [0.6787 - 12.1088] |
| Lentils intake (Cups/Day) | | | | | | | | |
| No | 61 | 7.63 | 11 | 4.78 | 40 | 10.25 | 0.439 | 0.0192 [0.2208 - 0.8748] |
| <2 | 715 | 89.48 | 214 | 93.04 | 339 | 86.92 | 2.012 | 0.0196 [1.1186 - 3.6195] |
| 2-5 | 21 | 2.62 | 3 | 1.30 | 11 | 2.82 | 0.455 | 0.2310 [0.1257 - 1.6495] |
| >5 | 2 | 0.25 | 2 | 0.86 | 0 | 0 | 8.544 | 0.1667 [0.4084 - 178.7760] |
| Egg intake (Pcs./Day) | | | | | | | | |
| **No** | **85** | **10.63** | **35** | **15.21** | **35** | **8.97** | **1.82** | **0.0189 [****1.1042 - 3.0016]** |
| <2 | 702 | 87.85 | 193 | 83.91 | 350 | 89.74 | 0.596 | 0.0348 [0.3687 - 0.9638] |
| 2-5 | 11 | 1.37 | 2 | 0.86 | 4 | 1.02 | 0.846 | 0.8481 [0.1538 - 4.6582] |
| >5 | 1 | 0.12 | 0 | 0 | 1 | 0.25 | 0.563 | 0.7256 [0.0228 - 13.8850] |
| Fish/Chicken intake (Pcs./Day) | | | | | | | | |
| No | 30 | 3.75 | 8 | 3.47 | 10 | 2.56 | 1.369 | 0.5141 [0.5326 - 3.5208] |
| <2 | 666 | 83.35 | 184 | 80 | 337 | 86.41 | 0.629 | 0.0363 [0.4076 - 0.9708] |
| 2-5 | 99 | 12.39 | 35 | 15.21 | 42 | 10.76 | 1.487 | 0.1063 [0.9187 - 2.4073] |
| >5 | 4 | 0.50 | 3 | 1.30 | 1 | 0.25 | 5.141 | 0.1573 [0.5316 - 49.7180] |
| Vegetables (Cups/Day) | | | | | | | | |
| No | 68 | 8.51 | 17 | 7.39 | 34 | 8.71 | 0.835 | 0.5618 [0.4557 - 1.5326] |
| <2 | 606 | 75.84 | 198 | 86.08 | 332 | 85.12 | 1.08 | 0.7434 [0.6782 - 1.7228] |
| 2-5 | 41 | 5.13 | 12 | 5.21 | 17 | 4.35 | 1.207 | 0.6253 [0.5662 - 2.5765] |
| >5 | 10 | 1.25 | 3 | 1.30 | 7 | 1.79 | 0.723 | 0.6409 [0.1851 - 2.8243] |
| Fruit intake (Daily) | | | | | | | | |
| Yes | 702 | 87.85 | 201 | 87.39 | 338 | 86.67 | 1.066 | 0.7959 [0.6554 - 1.7347] |
| No | 97 | 12.14 | 29 | 12.60 | 52 | 13.34 | 0.937 | 0.7959 [0.5765 - 1.5257] |
| Fried Food intake (Day/Week) | | | | | | | | |
| No | 210 | 26.28 | 55 | 23.91 | 116 | 29.74 | 0.742 | 0.1172 [0.5114 - 1.0777] |
| <2 | 195 | 24.40 | 101 | 43.91 | 160 | 41.02 | 1.125 | 0.4819 [0.8096 - 1.5646] |
| 2-5 | 347 | 43.42 | 59 | 25.65 | 93 | 23.84 | 1.101 | 0.6136 [0.7561 - 1.6058] |
| >5 | 47 | 5.88 | 15 | 6.52 | 21 | 5.38 | 1.225 | 0.5592 [0.6188 - 2.4285] |
| Carbonated drinks consumption (Days/Week) | | | | | | | | |
| No | 400 | 50.06 | 120 | 52.17 | 206 | 52.82 | 0.974 | 0.8762 [0.7031 - 1.3503] |
| <2 | 326 | 40.80 | 87 | 37.82 | 155 | 39.74 | 0.922 | 0.6364 [0.6599 - 1.2893] |
| 2-5 | 51 | 6.38 | 16 | 6.95 | 25 | 6.41 | 1.091 | 0.7915 [0.5700 - 2.0906] |
| >5 | 22 | 2.75 | 7 | 3.04 | 4 | 1.02 | 3.029 | 0.0797 [0.8770 - 10.4624] |
| Sweets intake (Days/Week) | | | | | | | | |
| **No** | **184** | **23.02** | **65** | **28.26** | **84** | **21.53** | **1.435** | **0.0591 [****0.9862 - 2.0881]** |
| <2 | 409 | 51.19 | 111 | 48.26 | 212 | 54.35 | 0.783 | 0.1423 [0.5650 - 1.0856] |
| 2-5 | 149 | 18.64 | 38 | 16.52 | 73 | 18.71 | 0.859 | 0.4910 [0.5585 - 1.3226] |
| >5 | 57 | 7.14 | 16 | 6.95 | 21 | 5.38 | 1.313 | 0.4260 [0.6710 - 2.5722] |
| Soy products intake (Days/Week) | | | | | | | | |
| No | 311 | 38.92 | 66 | 28.69 | 187 | 47.94 | 0.436 | < 0.0001 [0.3085 - 0.6187] |
| **<2** | **395** | **49.43** | **132** | **57.39** | **164** | **42.05** | **1.856** | **0.0002 [****1.3348 - 2.5812]** |
| **2-5** | **70** | **8.76** | **27** | **11.73** | **28** | **7.17** | **1.719** | **0.0560 [0.9863 - 2.9981]** |
| >5 | 23 | 2.87 | 5 | 2.17 | 11 | 2.82 | 0.765 | 0.6247 [0.2626 - 2.2320] |
| Curd/Yogurt intake (Days/Week) | | | | | | | | |
| No | 270 | 33.79 | 85 | 36.95 | 136 | 34.87 | 1.094 | 0.6006 [0.7799 - 1.5370] |
| <2 | 391 | 48.93 | 103 | 44.78 | 179 | 45.89 | 0.956 | 0.7877 [0.6891 - 1.3264] |
| 2-5 | 80 | 10.01 | 21 | 9.13 | 47 | 12.05 | 0.733 | 0.2623 [0.4263 - 1.2614] |
| >5 | 58 | 7.25 | 21 | 9.13 | 28 | 7.17 | 1.299 | 0.3855 [0.7195 - 2.3455] |
| Chocolate intake (Days/Week) | | | | | | | | |
| No | 177 | 22.15 | 49 | 21.30 | 98 | 25.12 | 0.806 | 0.2800 [0.5462 - 1.1912] |
| <2 | 317 | 39.67 | 98 | 42.60 | 146 | 37.43 | 1.240 | 0.2031 [0.8901 - 1.7297] |
| 2-5 | 212 | 26.54 | 59 | 25.65 | 102 | 26.15 | 0.974 | 0.8905 [0.6714 - 1.4136] |
| >5 | 92 | 11.51 | 24 | 10.43 | 44 | 11.28 | 0.916 | 0.7444 [0.5412 - 1.5509] |
| Processed foods (Days/Week) | | | | | | | | |
| No | 543 | 67.95 | 160 | 69.56 | 268 | 68.71 | 1.040 | 0.8256 [0.7309 - 1.4812] |
| <2 | 192 | 24.03 | 54 | 23.47 | 89 | 22.82 | 1.037 | 0.8510 [0.7055 - 1.5263] |
| 2-5 | 37 | 4.63 | 11 | 4.78 | 16 | 4.10 | 1.174 | 0.6889 [0.5352 - 2.5756] |
| >5 | 27 | 3.37 | 5 | 2.17 | 17 | 4.35 | 0.487 | 0.1637 [0.1774 - 1.3397] |
| Packaged fruit juice (Days/Week) | | | | | | | | |
| No | 467 | 58.44 | 136 | 59.13 | 244 | 62.56 | 0.865 | 0.3966 [0.6203 - 1.2083] |
| <2 | 259 | 32.41 | 70 | 30.43 | 118 | 30.25 | 1.008 | 0.9628 [0.7076 - 1.4373] |
| 2-5 | 55 | 6.88 | 18 | 7.82 | 23 | 5.89 | 1.354 | 0.3521 [0.7147 - 2.5682] |
| >5 | 18 | 2.25 | 6 | 2.60 | 5 | 1.28 | 2.062 | 0.2363 [0.6223 - 6.8355] |

**Supplementary Table 2** Degree of chemical exposure through synthetic cosmetics use

| **Variables** | **Total population**  **(N = 799)** | | **Individuals with diagnosed menstrual disorder**  **(N = 230)** | | **Individuals without diagnosed menstrual abnormality**  **(N = 390)** | | **Odds ratio (OR)** | ***p-*value [95% Confidence Interval]** |
| --- | --- | --- | --- | --- | --- | --- | --- | --- |
|  | **N** | **%** | **N** | **%** | **N** | **%** |  |  |
| Talcum powders (Days/Week) | | | | | | | | |
| <3 | 419 | 52.44 | 123 | 53.47 | 205 | 52.56 | 1.037 | 0.8257 [0.7484 - 1.4380] |
| 3-5 | 76 | 9.51 | 23 | 10 | 39 | 10 | 1.000 | 1.0000 [0.5809 - 1.7215] |
| >5 | 304 | 38.04 | 84 | 36.52 | 146 | 37.43 | 0.961 | 0.8199 [0.6859 - 1.3479] |
| Sunscreen lotions (Days/Week) | | | | | | | | |
| <3 | 429 | 53.69 | 121 | 52.60 | 208 | 53.34 | 0.971 | 0.8614 [0.7008 - 1.3463] |
| 3-5 | 96 | 12.01 | 36 | 15.65 | 43 | 11.02 | 1.497 | 0.0966 [0.9300 - 2.4113] |
| >5 | 272 | 34.04 | 73 | 31.73 | 139 | 35.64 | 0.839 | 0.3227 [0.5938 - 1.1873] |
| Body lotions (Days/Week) | | | | | | | | |
| <3 | 507 | 63.45 | 150 | 65.21 | 238 | 61.02 | 1.197 | 0.2977 [0.8530 - 1.6810] |
| 3-5 | 66 | 8.26 | 20 | 8.69 | 36 | 9.23 | 0.936 | 0.8224 [0.5282 - 1.6604] |
| >5 | 225 | 28.16 | 60 | 26.08 | 116 | 29.74 | 0.833 | 0.3296 [0.5783 - 1.2018] |
| Deodrants (Days/Week) | | | | | | | | |
| <3 | 489 | 61.20 | 132 | 57.39 | 222 | 56.92 | 1.019 | 0.9094 [0.7333 - 1.4169] |
| 3-5 | 130 | 16.27 | 45 | 19.56 | 62 | 15.89 | 1.286 | 0.2438 [0.8421 - 1.9664] |
| >5 | 180 | 22.52 | 53 | 23.04 | 106 | 27.17 | 0.802 | 0.2551 [0.5490 - 1.1724] |
| Lipsticks (Days/Week) | | | | | | | | |
| <3 | 714 | 89.36 | 195 | 84.78 | 351 | 90 | 0.619 | 0.0545 [0.3797 - 1.0093] |
| 3-5 | 71 | 8.89 | 32 | 13.91 | 31 | 7.94 | 1.871 | 0.0189 [1.1088 - 3.1592] |
| >5 | 14 | 1.75 | 3 | 1.30 | 8 | 2.05 | 0.631 | 0.4998 [0.1657 - 2.4029] |
| Body soaps (Days/Week) | | | | | | | | |
| <3 | 130 | 16.27 | 31 | 13.47 | 53 | 13.58 | 0.990 | 0.9687 [0.6150 - 1.5953] |
| 3-5 | 120 | 15.01 | 34 | 14.78 | 54 | 13.84 | 1.079 | 0.7469 [0.6788 - 1.7164] |
| >5 | 549 | 68.71 | 165 | 71.73 | 283 | 72.56 | 0.062 | < 0.0001 [0.0281 - 0.1402] |
| Shampoo (Days/Week) | | | | | | | | |
| <3 | 620 | 77.59 | 166 | 72.17 | 307 | 78.71 | 0.701 | 0.0649 [0.4811 - 1.0221] |
| 3-5 | 159 | 19.89 | 54 | 23.47 | 75 | 19.23 | 1.288 | 0.2088 [0.8677 - 1.9137] |
| >5 | 20 | 2.50 | 10 | 4.34 | 8 | 2.05 | 2.170 | 0.1078 [0.8441 - 5.5808] |
| Body oil (Days/Week) | | | | | | | | |
| <3 | 581 | 72.71 | 167 | 72.60 | 289 | 74.10 | 0.926 | 0.6838 [0.6413 - 1.3383] |
| 3-5 | 120 | 15.01 | 38 | 16.52 | 58 | 14.87 | 1.132 | 0.5834 [0.7253 - 1.7696] |
| >5 | 98 | 12.26 | 25 | 10.86 | 43 | 11.02 | 0.984 | 0.9521 [0.5837 - 1.6592] |
| Hair oil (Days/Week) | | | | | | | | |
| <3 | 356 | 44.56 | 108 | 46.95 | 182 | 46.67 | 1.011 | 0.9443 [0.7299 - 1.4024] |
| 3-5 | 240 | 30.03 | 76 | 33.04 | 112 | 28.71 | 1.225 | 0.2580 [0.8618 - 1.7410] |
| >5 | 202 | 25.28 | 46 | 20 | 96 | 24.61 | 0.765 | 0.1871 [0.5148 - 1.1386] |

**Supplementary Data S3** Structured Questionnaire

**Structured Questionnaire for Women**

CODE: [OFFICE USE ONLY] DATE: ___________

Full Name: ______________________________________________________________

DOB: _________________ Age: ___________ Educational Status: _________________

Name of Father: __________________________________ Education: _________________

Occupation: ____________________ Family income (monthly): Less than/ equal Rs.15000 Rs.15,000 – Rs.30,000 Rs.30,000 –Rs.50,000 more than Rs.50,000

Religion: Hindu Muslim Christian Others___________

Address: ___________________________________________________________________ Contact No.________________________________________________________

General Examination: Height (cm):________Weight (kg):_______ Waist (cm): _______ Hip (cm): ________ Blood pressure: ____________ Pulse rate: _________

**Food Habit:**

Daily water consumption: _______ litre If Any addiction (for adults): _________________

Tea consumption: _______ Cups Liquor With Milk With Sugar

Coffee consumption: ________Cups Black With Milk With Sugar

| Foods | How many days in a month? | How many days in a week? | How many in a day? |
| --- | --- | --- | --- |
| Rice (Cups) |  |  |  |
| Roti (Pcs.) |  |  |  |
| Lentil (Cups) |  |  |  |
| Fish (Pcs.) |  |  |  |
| Chicken (Pcs.) |  |  |  |
| Egg (Pcs.) |  |  |  |
| Vegetables (Cups) |  |  |  |
| Fruits (Pcs.) |  |  |  |
| Fried food/chips |  |  |  |
| Soft drinks |  |  |  |
| Sweets (Pcs.) |  |  |  |
| Soy product |  |  |  |
| Curd |  |  |  |
| Candy/Chocolate |  |  |  |
| Cottage cheese |  |  |  |
| Processed food (ham, sausage, etc.) |  |  |  |
| Package food/juice |  |  |  |

**Cosmetics used:**

| Items | How many days in a month? | How many days in a week? | How many in a day? | Which brand? |
| --- | --- | --- | --- | --- |
| Talcom powder |  |  |  |  |
| Sunscreen lotion |  |  |  |  |
| Moisturizer |  |  |  |  |
| Deodorant |  |  |  |  |
| Lipstick |  |  |  |  |
| Make-up blusher |  |  |  |  |
| Sindoor |  |  |  |  |
| Body soap |  |  |  |  |
| Shampoo |  |  |  |  |
| Body oil |  |  |  |  |
| Hair oil |  |  |  |  |

**Physical Exercise:**

| Type | Duration(min) | | | | Days/week | | | Years | | |
| --- | --- | --- | --- | --- | --- | --- | --- | --- | --- | --- |
|  | <30 | 30-60 | >60-120 | 1-3 | | 4-5 | 6-7 | 1 | 2-5 | >5 |
| Walking |  |  |  |  | |  |  |  |  |  |
| Jogging |  |  |  |  | |  |  |  |  |  |
| Dance |  |  |  |  | |  |  |  |  |  |
| Yoga |  |  |  |  | |  |  |  |  |  |
| Sports |  |  |  |  | |  |  |  |  |  |

Watching TV: ____ (Hour/Day) Mobile____ (Hour/day) Computer/Laptop ____ (Hour/Day) Duration of Resting period/leisure time (Hours): __________ Sleeping (Hours): ______ (from ______ to ______) Duration of working (Hours):_______ (from ______ to ______) Journey time (Hours):__________

Mode of transport frequently availed: _________________________

**Health problems: [Y/N/NA]**

Menstrual cycle started? _______ ; (if yes) Days of interval it happen _______ (eg. 28 days normally) ; Age at when first started _____________ ; Period lasts for ______days ; Do you feel abdominal cramps/extreme lower back pain? _______________________

Amount of menses: Low Moderate Heavy

Do you use Sanitary napkins? Yes No

Do you have thyroid problem? Yes No NA

Do you have Gastro-intestinal problem (Indigestion/Ulcer etc.)? Yes No NA

Do you feel Drooling/Dizziness/Tiredness? Yes No NA

Do you have Headache/Bodyache very frequently? Yes No NA

Do you have Anaemia? Yes No NA

Do you have Arthritis? Yes No NA

Do you have Diabetes? Yes No NA

Do you have PCOS? Yes No NA

Do you have Endometriosis? Yes No NA

History of congenital/any major illness: ______________________________________

**Parental Details:**

| **Disease** | **Yes/No** | **Relation with you** |
| --- | --- | --- |
| Hypertension |  |  |
| Cardiovascular disease |  |  |
| Pulmonary disease |  |  |
| Thyroidism |  |  |
| Anaemia |  |  |
| Arthritis |  |  |
| Diabetes |  |  |
| Gynaecological problems |  |  |

ARE YOU WILLING TO PROVIDE **MOUTH-WASH/URINE/BLOOD** OF YOURS FOR RESEARCH [Y/N]

Consent: I am participating as a volunteer in the research program. The information given will not reveal identity and be used for research purpose.

Signature: _______________________ date_____________
